# Expansion of ribosomally produced natural products: a nitrile hydratase- and Nif11-related precursor family

**DOI:** 10.1186/1741-7007-8-70

**Published:** 2010-05-25

**Authors:** Daniel H Haft, Malay Kumar Basu, Douglas A Mitchell

**Affiliations:** 1 The J Craig Venter Institute, 9704 Medical Center Drive, Rockville, MD 20850, USA; 2 Department of Chemistry, University of Illinois at Urbana-Champaign, Urbana, IL 61801, USA; 3 Department of Microbiology, University of Illinois at Urbana-Champaign, Urbana, IL 61801, USA; 4 Institute for Genomic Biology, University of Illinois at Urbana-Champaign, Urbana, IL 61801, USA

## Abstract

**Background:**

A new family of natural products has been described in which cysteine, serine and threonine from ribosomally-produced peptides are converted to thiazoles, oxazoles and methyloxazoles, respectively. These metabolites and their biosynthetic gene clusters are now referred to as thiazole/oxazole-modified microcins (TOMM). As exemplified by microcin B17 and streptolysin S, TOMM precursors contain an N-terminal leader sequence and C-terminal core peptide. The leader sequence contains binding sites for the posttranslational modifying enzymes which subsequently act upon the core peptide. TOMM peptides are small and highly variable, frequently missed by gene-finders and occasionally situated far from the thiazole/oxazole forming genes. Thus, locating a substrate for a particular TOMM pathway can be a challenging endeavor.

**Results:**

Examination of candidate TOMM precursors has revealed a subclass with an uncharacteristically long leader sequence closely related to the enzyme nitrile hydratase. Members of this nitrile hydratase leader peptide (NHLP) family lack the metal-binding residues required for catalysis. Instead, NHLP sequences display the classic Gly-Gly cleavage motif and have C-terminal regions rich in heterocyclizable residues. The NHLP family exhibits a correlated species distribution and local clustering with an ABC transport system. This study also provides evidence that a separate family, annotated as Nif11 nitrogen-fixing proteins, can serve as natural product precursors (N11P), but not always of the TOMM variety. Indeed, a number of cyanobacterial genomes show extensive N11P paralogous expansion, such as *Nostoc*, *Prochlorococcus *and *Cyanothece*, which replace the TOMM cluster with lanthionine biosynthetic machinery.

**Conclusions:**

This study has united numerous TOMM gene clusters with their cognate substrates. These results suggest that two large protein families, the nitrile hydratases and Nif11, have been retailored for secondary metabolism. Precursors for TOMMs and lanthionine-containing peptides derived from larger proteins to which other functions are attributed, may be widespread. The functions of these natural products have yet to be elucidated, but it is probable that some will display valuable industrial or medical activities.

## Background

Bacteriocins are polypeptide-based natural products of ribosomal origin, usually functioning as antibiotics toxic to rival strains or species of bacteria [1]. Peptide products resembling the bacteriocins in their size, precursor sequence, posttranslational modifications and co-clustering with maturation enzymes occasionally prove to have a signalling function or other non-antibiotic activity [2]. Collectively, these products represent a large reservoir of molecules with vast potential. Bacteriocin production and resistance mechanisms are, without question, major contributors to microbial ecology dynamics. Despite decades of research, including extensive work on low molecular weight bacteriocins (microcins), these processes are little understood. The small size and unusual amino acid composition of microcin precursor peptides hinder even the recognition of the open reading frame (ORF) as the coding region of a real gene [3,4]. Furthermore, the low level of sequence similarity often found even among microcins of the same general class impedes identification of new microcins by sequence similarity. These arguments represent possible explanations for the reason why the study of ribosomally-produced peptide natural products has lagged behind that of the well-known non-ribosomal peptide synthetase and polyketide synthase systems [5,6].

**Figure 1:**
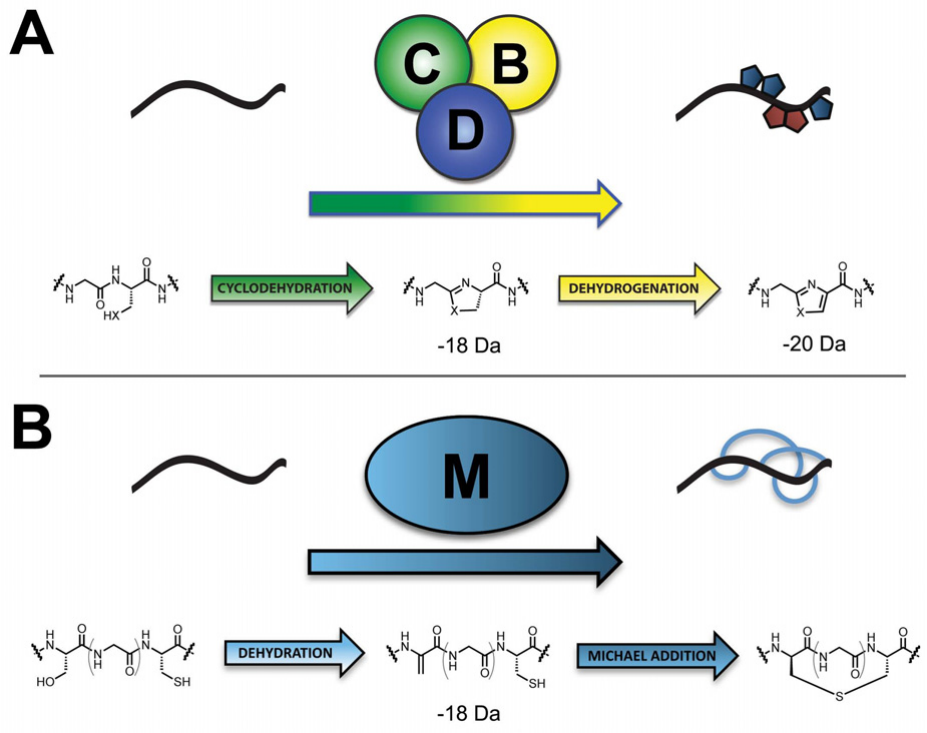
**The biosynthesis and defining chemical features of TOMM and lanthionine-containing natural products**. (A) Through the action of a trimeric 'BCD' complex, consisting of a cyclodehydratase (green), dehydrogenase (yellow) and docking/scaffolding protein (blue), thiazoles and (methyl/oxazoles are incorporated onto a peptidic scaffold (black). These heterocycles are synthesized from serine/threonine (X = O) and cysteine (X = S) residues of an inactive precursor peptide and yield a bioactive natural product. The chemical transformations carried out by the cyclodehydratase the dehydrogenase are shown, along with the corresponding mass change in Daltons. (B) A bifunctional synthase, LanM, catalyzes both the dehydration and Michael-type addition steps required to synthesize lanthionine crosslinks.

A subset of microcins has been recently described in which the amino acid side chains of cysteine, serine and threonine from a ribosomally produced precursor undergo heterocyclization to generate a product with thiazole or (methyl)oxazole moieties. These include trichamide [7], the patellamides [8], goadsporin [9] and microcin B17 [10], among others. Building on these earlier studies, a research team led by Jack Dixon [4] described three types of proteins that represent a conserved biosynthetic machine for the formation of these heterocycle-containing metabolites across numerous microbial phyla. A zinc-tetrathiolate containing cyclodehydratase, flavin mononucleotide-dependent dehydrogenase and a docking scaffold protein are collectively responsible for the installation of thiazole and (methyl/oxazole modifications to a peptide precursor (Figure 1A). In each case studied so far, the cyclodehydratase, dehydrogenase and docking scaffold proteins form a trimeric complex (BCD) and serve to convert inactive, unstructured peptides into bioactive natural products [4,11,12]. The thiazole/oxazole heterocycles are biosynthesized over two distinct chemical transformations. The first is catalyzed by the cyclodehydratase (C), which converts Cys and Ser/Thr residues into the corresponding thiazoline and (methyl)oxazoline with loss of water from the amide backbone. In a second reaction, the dehydrogenase (B) removes two electrons and two protons to afford the aromatic thiazole and (methyl)oxazole [10,13]. The docking scaffold protein (D) appears to play a role in trimer assembly and the regulation of enzymatic activity. For each oxidized heterocycle formed, 20 Da is lost from the parent peptide, which provides a convenient measure of product formation by mass spectrometry (Figure 1) [4,8,14]. This class of natural product has been termed the thiazole/oxazole-modified microcins (TOMMs).

In a simplified view, the purpose of the TOMM biosynthetic machinery is to recognize substrate and install structural constraints that restrict peptide bond rotation, thus endowing the modified peptide with a rigidified tertiary structure. By restricting conformational flexibility at the correct locations, the altered steric and electronic properties of the molecule, in conjunction with the physiochemical properties of the adjacent amino acids, lead to a specific biological activity. This type of rationale could also be extended to another family of post-translationally modified peptides, the lantibiotics, with the only major differences being the chemical composition (lanthionine containing) and biosynthetic installation of the structural constraints (Figure 1B) [15,16].

Again, similar to the lanthionine-containing peptides (lantipeptides), TOMM precursor peptides are bipartite: they contain an N-terminal leader sequence and a C-terminal 'core' peptide. The leader sequence has been shown in several cases to be critical to substrate recognition by the modifying enzymes, while the core peptide serves as a foundation upon which the active molecule is built [11,17-19]. Outside of the leader region, TOMM precursors tend to be rich in heterocyclizable residues (Cys, Ser, Thr) and also in Gly, whose minimal side chain reduces the energetic barrier required for cyclodehydration. Clues that support the interpretation of an ORF as a TOMM precursor include sequence similarity to previously identified TOMM precursors, a leader peptide cleavage motif, and a hypervariable C-terminal core region rich in Gly, Cys, Ser and Thr [4,11]. Also aiding the identification of a TOMM cluster is the tendency of the modification enzymes to cluster with other genes necessary for the complete chemical maturation, export and immunity to the natural product [4,20,21]. Identification of genes encoding enzymes involved in lanthionine formation [15,22,23], dehydroalanine production [9], peptide macrocyclization [7,8,24,25] and thiazole/oxazole synthesis provide anchoring information for annotating post-translationally modified peptide biosynthetic clusters, such as the TOMMs and lantipeptides. Identification of other proteins (for example dehydrogenases, acetyltransferases, methyltransferases, proteases and transporters) in the local genomic region do not necessarily mark a biosynthetic cluster on their own but instead, help to define the extent and complexity of a proposed cluster [4].

Recent TOMM precursor identification by several groups [3,8,24,26-29], including ours [4,30], provide a growing number of short leader peptide sequences, a few of which show a moderate level of similarity with one another. However, many of the apparent TOMM biosynthetic systems have remained orphan systems, in that the thiazole/oxazole forming genes (encoding for the BCD synthetase complex, Figure 1A) could be detected but the TOMM precursors themselves could not be found. The current availability of well over 1000 complete bacterial and archaeal genomes permits the use of comparative genomics methods to locate the substrates for orphan TOMMs while simultaneously broadening the search for previously unknown families of post-translationally modified peptides. Our results illustrate the power of applying multiple informatics tools to the analysis of large numbers of fully sequenced genomes and suggest new opportunities to identifying secondary metabolite biosynthetic systems.

## Results and discussion

Using a combination of informatics tools against a large number of sequenced genomes, we discovered several protein families that appear to represent an entirely new class of post-translationally modified peptide. The precursors have uncharacteristically long leader sequences and large paralogous family counts per genome. Analysis of the local genomic region predicts that these precursors will have variable chemical fates, including thiazole/oxazole and lanthionine formation. These families, surprisingly, include one set of sequences with strong similarity to the alpha subunit of the enzyme nitrile hydratase (NHase) [31,32] while another set exhibits striking similarity to nitrogen-fixing proteins from cyanobacteria (Nif11) [33,34].

### Description of NHase-related leader microcin family

**Figure 2:**
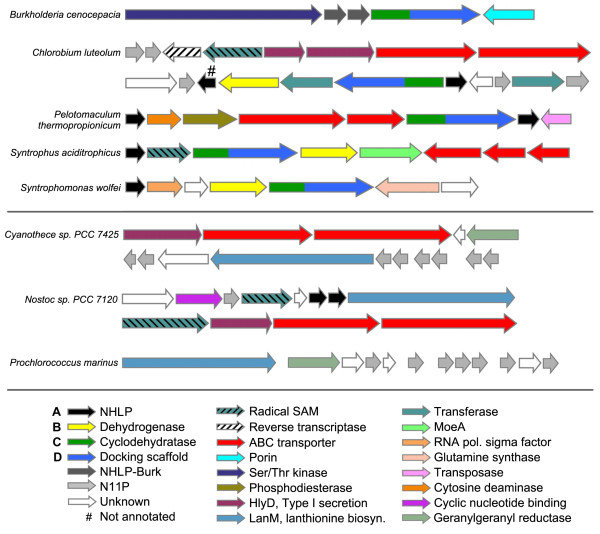
**The genetic organization of TOMM and lanthionine biosynthetic clusters that utilize NHase- and Nif11-related precursor peptides**. Genomic regions are shown from selected organisms in which the precursor peptides are clustered with the cognate modification enzymes. In most cases, a transport system is also visible in the local region. The TOMM precursors represented by *Burkholeria cenocepacia *(dark gray ORFs, NHLP-Burk) are accompanied by a large, Ser/Thr kinase. Highly similar clusters have been identified in *Acidovorax avenae *and *Delftia acidovorans*. In several species, precursors shown in black (NHLP) and light grey [Nif11-derived peptide (N11P)] may cluster with each other as well as with other modification and transporter genes. *Note*: only those precursors closest to the cyclodehydratase-docking scaffold protein or LanM-like lanthionine synthase are shown. For instance, *Pelotomaculum thermopropionicum *has 12 NHLPs (only two are shown) and seven N11P family precursors, while *Cyanothece *sp. PCC 7425 and *P. marinus *have 18 and 29 predicted N11P precursors, respectively (eight and seven are shown). Transport proteins, including those homologous to HlyD (type I secretion, purple) and ABC transporters (red), correspond to the transport genes detected by PPP.

One family of the newly discovered precursor peptides is described by TIGRFAMs model TIGR03793 (Table 1) and designated NHLP (nitrile hydratase-related leader peptides - as described below). In five diverse bacterial species, spanning several phyla, including firmicutes (*Syntrophomonas wolfei *subsp. *wolfei *str. Goettingen, *Pelotomaculum thermopropionicum *SI), proteobacteria (*Stigmatella aurantiaca *DW4/3-1, *Syntrophus aciditrophicus *SB [35]) and the chlorobi group (*Chlorobium luteolum *DSM 273), NHLP precursors are found adjacent to a cyclodehydratase-docking scaffold fusion protein (TIGR03882), a required component of TOMM biosynthesis [4]. The local genomic context of four of these biosynthetic clusters is illustrated in the upper portion of Figure 2. Additional species provide further supporting evidence for a link between NHLP and the cyclodehydratase-docking scaffold by co-occurrence within the same genome. Although not in close proximity to the cognate NHLP substrate, the cyclodehydratase-docking scaffold proteins from *Microscilla marina *ATCC 23134 (chlorobi group) and *Methylobacterium sp*. 4-46 (proteobacteria) represent two examples of this genetic organization. Akin to the NHLP system, previous informatics work has shown that the *Bacillus anthracis *and *B. cereus *TOMM precursors are encoded more than one megabase away from the modification cluster [30]. As with the NHLPs from *M. marina *and *Methylobacterium*, recognizing an orthologous cluster in *B. licheniformis*, in which all components were clustered, accelerated the identification of the precursors in *B*. *anthracis *and other members of the *B. cereus *group.

**Table 1:** Description of TIGR models

Accession	Colour*	Description	Abbreviation
TIGR01323	N/A	Nitrile hydratase, alpha subunit	NHase

TIGR03603	N/A	Cyclodehydratase (single ORF)	C

TIGR03604	Blue	Docking scaffold	D

TIGR03882	Green	Cyclodehydratase-docking (fused)	CD

TIGR03605	Yellow	Dehydrogenase	B

TIGR03793	Black	NHase-related leader peptide	NHLP

TIGR03795	Dark gray	*Burkholderia *NHLP	NHLP-Burk

TIGR03798	Light gray	Nif11-related peptide	N11P

TIGR03794	Purple	HlyD-like, type I secretion	Trans-Fuse

TIGR03796	Red	ABC transporter with peptidase	Trans-Cleave

TIGR03797	Red	ABC transporter	Trans

*Colour coded in Figure 2.

**Figure 3:**
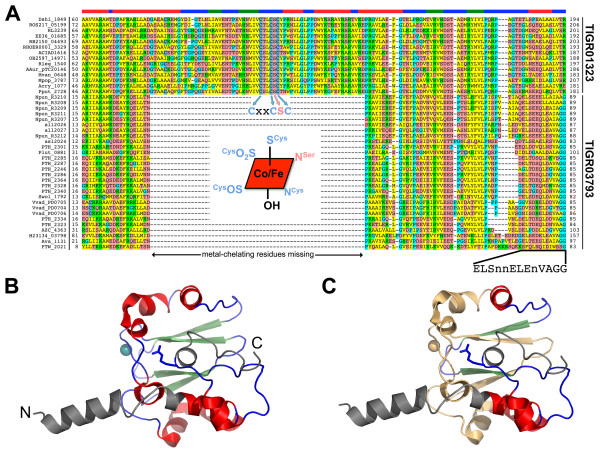
**Alignment of nitrile hydratase (NHase) with nitrile hydratase leader peptide (NHLP) sequences**. (A) Fourteen members of the NHase alpha subunit protein family (TIGR01323), identified by locus tags, are shown aligned to the leader sequences of 28 members of the NHLP family (TIGR03793). Along the top of the figure is a colour-coded region depicting the anticipated secondary structure for that region (red, alpha-helix; blue, loop; green, beta-sheet). Relative to NHase, the NHLP sequences exhibit a 63-residue deletion that carries the residues required for iron/cobalt ligation, the CxxCSC motif. Without the ability to bind the required catalytic metal, the truncation seen in NHLP is presumed to abolish NHase activity. Shown in the truncated region is the canonical metal coordination architecture, with two of the three Cys thiol ligands being oxidized to sulphenic and sulphinic acids. Also shown is the putative leader sequence cleavage site for NHLP, which is not conserved with full-length NHase. (B) Crystal structure of the NHase from *Bacillus smithii*, the most closely related NHase to the NHLP family with a known structure [38]. The N- and C-termini have been labelled, the metal centre is shown as a cyan sphere and the beta subunit has been omitted for clarity. The colour coding is by secondary structure as in panel A with the addition of the N- and C-terminal extensions (grey) that are not included in the alignment. The final residue of the NHase alignment, Arg, is shown in blue stick format. (C) Same as in panel B, but with the insertion-deletion region omitted in NHLP's shown in wheat. This figure was generated using a previously described, web-based program [36] and PyMOL.

An unmistakable feature of the NHLP family is its close sequence similarity to the alpha subunit of NHase, which is described by TIGR01323 (Table 1 and Figure 3). Previously characterized NHases (EC 4.2.1.84) are composed of two subunits, alpha and beta, which together catalyze the general reaction shown below [31,32].(1)

For over 90% of genomes containing a member of the NHase family (TIGR01323), that member occurs as the highest scoring sequence in the genome to a search using the fragment version (local-local scoring) Hidden Markov Model (HMM) of TIGR03793. Fragment model searches are preferred when match regions do not span the full length of the seed alignment or the target sequence. This is certainly the case when comparing sequences that have either a large insertion or deletion (*indel*) relative to each other. The median E-value for these HMM genome search results is 1e-7, despite the short length (82 amino acids) of the TIGR03793 model. As these sequences are neither repetitive nor low in complexity in the regions covered by the HMM, the consistently low E-values for alignment between the two families predicts substantial sequence similarity between NHases and NHLPs. Furthermore, over three-quarters of the hits from TIGR03793 (fragment model) to NHases found two match segments, straddling a large *indel *region present in the alpha subunit of NHase, but not in TIGR03793 family sequences. The above described similarity and *indel *are clearly evident in the alignment [36] shown in Figure 3A. The sequences align convincingly over approximately 20 residues N-terminal, and 50 residues C-terminal, to the region deleted from the NHLP family.

**Figure 4:**
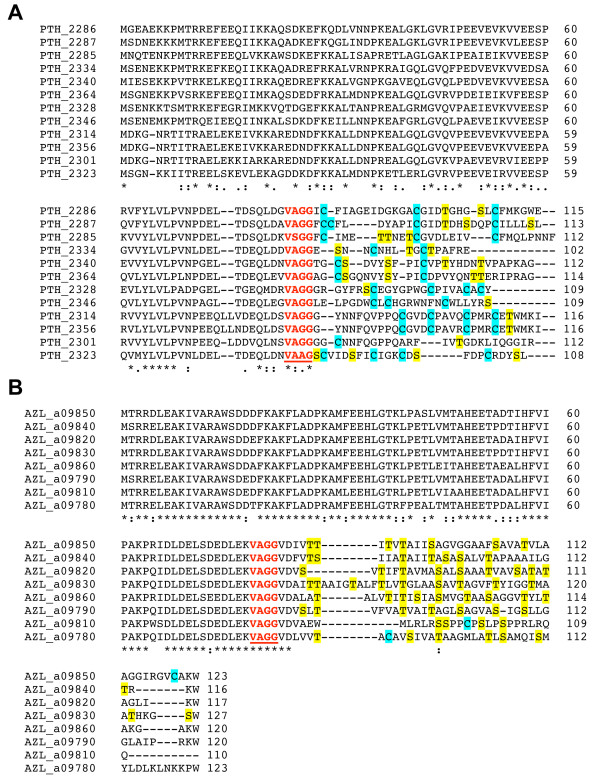
**Sequence alignment of nitrile hydratase leader peptides (NHLPs) from natural combinatorial biosynthetic clusters**. ClustalW alignment [44] of NHLPs from a putative thiazole/oxazole-modified microcin cluster from (A)*Pelotomaculum thermopropionicum *SI (12 sequences) and (B) *Azospirillum *sp. B510 (eight sequences). Possible sites for thiazole formation are highlighted in cyan and sites of potential oxazole and methyloxazole formation are yellow. The locus tag is given to the left of the sequence and the amino acid position is given on the right. An asterisk implies an invariant residue, while the semicolon and period show positions that are highly and moderately related, respectively. Underlined red text indicates the putative leader peptide cleavage motif.

The deleted region includes the NHase CxxCSC motif, in which two of the three invariant cysteines are oxidatively modified - one to sulphenic acid, the other to sulphinic acid. Together, with a reduced cysteine thiol and amide nitrogen of serine, these moieties serve as ligands for the catalytic metal centre (Figure 3). NHase enzymes use either a non-heme iron or a non-corrinoid cobalt metal ion to activate water for hydrolyzing nitriles to amides [31,32,37] (Equation 1). As all NHLPs lack the entire active site region, they are suspected of being devoid of NHase enzymatic activity. Supporting this is a visual depiction of the segment of NHase missing in NHLP, provided by the X-ray crystal structure of the NHase from *B. smithii *(Figure 3B-3C) [38]. Another key difference between the NHase and NHLP families is the observation that the NHLPs harbour a classic leader peptide cleavage site (Gly-Gly), which occurs at the extreme C-terminal end of the region of similarity between the TIGR01323 and TIGR03793 models (Figure 3A). This motif also marks the end of sequence conservation among members within TIGR03793. Following the Gly-Gly motif is a hypervariable region, in which many sequences are rich in residues that are targeted by posttranslational modifying enzymes (Cys, Ser, Thr, Figure 4). This composition suggests that the hypervariable region is the 'core peptide' and the homologous region comprises the leader sequence [18].

### Phylogenetic profiling studies show connection to a putative microcin export system

We computationally evaluated the candidacy of the NHLP family as post-translationally modified peptide precursors by the method of partial phylogenetic profiling (PPP) [39], in which the profile serves as a query against an entire genome. A phylogenetic profile was constructed on the basis of whether or not each sequenced bacterial and archaeal genome carries a NHLP. Using PPP, all sequences in the genome were evaluated to determine which best match the profile. In a collection of 1450 complete, or nearly complete, microbial genomes, NHLPs occur in 14 species. Within these 14, each contain between one and 12 copies of NHLP in their respective genome (Figures 2, 3, 4). As shown in Table 2, the phylogenetic profile of these 14 NHLPs identified a three-gene ABC transport cluster as the only high-scoring protein family other than the NHLP precursor gene itself. The top hits from our PPP search include families TIGR03794 (TransFuse), TIGR03796 (TransCleave), and TIGR03797 (TransABC), which are also described in Table 1. A notable difference between TIGR03796 and TIGR03797 is that while both contain the adenosine triphosphate (ATP)-binding cassette domain and permease domain, the latter lacks the peptidase domain. TIGR03794 resembles the HlyD membrane fusion protein of type I secretion systems, suggesting a role in transport across the outer membrane. Natural product export, including unmodified and modified peptides, is often attributed to a nearby ABC transport system that combines a protease domain, permease domain and ATP-binding cassette, either as multiple ORFs or as a single polypeptide sequence [40,41]. The purpose of such a cassette is to simply cleave the leader peptide and export the mature product from the cell. NHLPs are adjacent to these transporter cassettes in a diverse array of bacterial species, including: *Nostoc *sp. PCC 7120* (cyanobacteria), *Anabaena variabilis *ATCC 29413 (cyanobacteria), *M. marina *ATCC 23134 (chlorobi group), *C. luteolum *DSM 273* (chlorobi group), *Victivallis vadensis *ATCC str. BAA-548 (chlamydia group), and *P. thermopropionicum *SI* (firmicutes, * denotes a cluster shown in Figure 2). It is important to note that not all of putative biosynthetic clusters identified next to the ABC transporter genes are adjacent to TOMM machinery. In the case of *Nostoc *sp. PCC 7120, the NHLP and ABC transporter genes are adjacent to an enzyme resembling LanM, which is involved in lanthionine biosynthesis [16,42] (Figure 2). The findings from PPP strongly support our interpretation of NHLPs as post-translationally modified peptide precursors and further argue that many, if not all, NHLP peptides will be subjected to leader peptide cleavage upon export.

**Table 2:** Partial phylogenetic profiling (PPP) results

*Chlorobium luteolum*					
78186745	8	8	16	-16.124	NHLP
78186739	9	19	26	-13.212	Trans
78186738	9	22	29	-12.492	Trans-Cleave
78186736	8	16	17	12.045	Trans-Fuse
78187852	10	95	123	-7.475	(PAS domain)
					
*Nostoc *sp. PCC 7120					
**17229519**	**9**	**9**	**23**	**-18.140**	**NHLP**
**17229518**	**11**	**21**	**38**	**-16.662**	**NHLP**
**17229516**	**8**	**8**	**23**	**-16.124**	**NHLP**
**17233313**	**10**	**20**	**26**	**-14.927**	**Trans**
**17229512**	**10**	**20**	**26**	**-14.927**	**Trans**
**17229513**	**11**	**33**	**42**	**-13.969**	**Trans-Cleave**
**17233311**	**10**	**25**	**31**	**-13.698**	**Trans-Cleave**
**17229514**	**9**	**24**	**33**	**-12.080**	**Trans-Fuse**
**17233314**	**9**	**26**	**35**	**-11.709**	**Trans-Fuse**
17228094	7	19	27	-9.451	(S-layer homol.)
					

*Microscilla marina *ATCC 23134					
**123986279**	**9**	**16**	**23**	**-14.108**	**Trans**
**123988060**	**9**	**21**	**28**	**-12.717**	**Trans-Cleave**
**123988059**	**7**	**10**	**14**	**-12.041**	**Trans-Fuse**
123992175	8	29	54	-9.570	(HAMP domain)
					

*Victivallis vadensi*s ATCC BAA-548					
**150259686**	**8**	**10**	**16**	**-14.479**	**Trans-Fuse**
**150259687**	**9**	**17**	**24**	**-13.784**	**Trans-Cleave**
**150259688**	**8**	**13**	**20**	**-13.033**	**Trans**
**150259679**	**6**	**6**	**15**	**-12.093**	**NHLP**
**150259681**	**5**	**6**	**8**	**-9.303**	**NHLP**
**150259680**	**4**	**4**	**6**	**-8.062**	**NHLP**
150257768	10	88	119	-7.798	(GAF domain)

This table shows the results of PPP, where the profile contains 14 'YES' genomes having proteins recognized by TIGR03793, about 1% of genomes and 1437 'NO' genomes. PPP scores each protein by selecting a BLAST score cutoff that gives the best possible fit between YES genomes in the profile and the set of genomes in the BLAST hits list, then scoring the fit at that depth. Columns, from left to right, are gi number, number of YES genomes encountered at the optimal depth, the total number of genomes at that depth, and the number of proteins at that depth (which can differ from the total number of genomes because several proteins may come from the same genome), the PPP score (a negative logarithm from the binomial distribution), and the protein family abbreviation. As PPP scores are not corrected for taxonomic relationships between species, scores are for comparison within each genome only and are shown down to the first noise hit. Results are shown in boldface except for noise hits. *Note: Microscilla marina *ATCC 23134 contains the nitrile hydratase leader peptide (NHLP) protein, 123988058, which is not detected by PPP. This gene is found co-clustered with the transport proteins identified by PPP, as shown.

The fact that correlation to a transport cassette emerges from PPP as a stronger relationship to the NHLP family, rather than any posttranslational tailoring enzyme, argues that the conservation in the leader peptide reflects a common mechanism of handling by the transport system (Table 2). The transport system appears to be providing more evolutionary pressure in order to maintain sequence similarity in this region than interaction with modification enzymes, which are usually considered to be highly specific [11,18,19]. This finding suggests a mix-and-match evolutionary pattern for post-translationally modified peptide biosynthesis and export systems, in which similarity in the leader peptide region provides only indirect evidence of which class of modification (thiazole/oxazole versus lanthionine) will occur. The broader species distribution of the newly defined putative export system, relative to the NHLP family through which they were detected, provides a unique opportunity to discover additional post-translationally modified peptides families in emerging and existing genomes.

### Core peptide hypervariability and natural combinatorial biosynthesis

**Figure 5:**
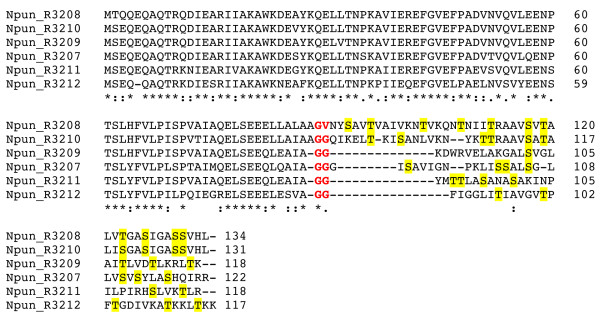
**Sequence alignment of nitrile hydratase leader peptides (NHLPs) from *Nostdoc punctiforme *PCC 73102**. Shown is a ClustalW alignment of six selected NHLP substrates. *N. punctiforme *PCC 73102 has at least 16 total substrates, half of which are NHLP and the other half N11P. The coloring scheme and notation are identical to Figure 4.

The hypervariability observed in NHLPs after the Gly-Gly motif is reminiscent of the variability in the core peptides of experimentally validated antimicrobial peptides, such as lichenicidin [42] and mersacidin [43]. An illustration of NHLP hypervariability is shown in Figure 4, where members of TIGR03793 are aligned using ClustalW [44]. Intriguingly, all 12 substrates shown in panel A are from the same organism, *P. thermopropionicum *SI, a thermophilic, clostridia class bacterium [45], while all eight members shown in panel B are from *Azospirillum *sp. B510, a proteobacterial rice endophyte [46]. Within the local genomic context of the NHLPs from *Azospirillum*, there are a LanM-like lanthionine-forming enzyme (AZL_a09720) and an unfused docking scaffold protein (AZL_a09740). While these two genes are plasmid-borne, an additional copy of the unfused docking scaffold protein (AZL_022710) can be found on the chromosome, along with a cyclodehydratase encoded 3 ORFs away (AZL_022680). Therefore, it is not possible to determine the chemical fate of the *Azospirillum *peptide precursors at this time (Figure 4B). A more straightforward case is demonstrated with *P*. *thermopropionicum*, which contains one TOMM biosynthetic cassette and no discernable lanthionine-forming enzymes (Figure 2). This implies that the single *P*. *thermopropionicum *cyclodehydratase-docking scaffold fusion protein will process all of the NHLPs into 12 distinct natural products (Figure 4A). Supporting this is the observation that the leader peptide region is highly conserved. The leader sequence of post-translationally modified peptides typically contains specific binding motifs recognized by the modifying enzymes [11,17,18]. This permits the selective modification of the desired peptide in a complex environment, such as the bacterial cytosol. Given that the *P*. *thermopropionicum *genome is relatively small (3.0 megabase, 2930 coding sequences), if this organism is to produce an extensive array of secondary metabolites, it must do so in a highly genome-efficient manner. This is in contrast to the much larger genome sizes of organisms renowned for secondary metabolism, such as *Streptomyces coelicolor *(8.7 megabase, 7825 coding sequences) [47]. Such examples of natural combinatorial biosynthesis are becoming more frequent, as demonstrated with the cyanobactins by Eric Schmidt's group [24]. It appears that natural combinatorial biosynthesis could be an underappreciated trait of cyanobacteria, given that eight NHLPs were also identified in *Nostoc punctiforme *PCC 73102 (Figure 5 shows an alignment of six of these).

### NHLPs from Burkholderia

Members of a second putative microcin precursor family, TIGR03795 (Table 1), occur near cyclodehydratase-docking scaffold fusion proteins in many proteobacteria of the *Burkholderia *order, including *Delftia acidovorans *SPH-1, two subspecies of *Acidovorax avenae *and multiple members within the genus *Burkholderia*: *B. cenocepacia, B. ambifaria, B. pseudomallei*, *B. thailandensis*, *B. oklahomensis *and *B. mallei *[48-50]. TIGR03795 family sequences occur exclusively as tandem gene pairs in the *Burkholderia *genus, suggesting these may form a two-peptide product, which are well-known (Figure 2) [41]. The tandem pairs in *D. acidovorans *and *A. avenae *are fused to yield a single polypeptide, further suggesting that the separate peptides from the *Burkholderia *genus function together. One member of each *Burkholderia *NHLP (NHLP-Burk) pair contains either Cys-Cys, or a single Cys, as the C-terminus (for example, Bcen_5137 and Bcen_5138). Members of this family were discovered as the top hits in their respective genomes to TIGR03793, suggesting a sequence relationship to the NHLP substrates described above. An alignment of NHase, NHLP and NHLP-Burk reveals a moderate level of sequence similarity. Relative to NHLP and NHase, NHLP-Burk contains an insert of about 15 amino acids N-terminal to a Pro-Xaa-Xaa-Pro motif conserved amongst the three families. A major difference between NHLP and NHLP-Burk lies in the leader peptide cleavage region (Table 3).

**Table 3:** Motif relationships in nitrile hydratase leader peptide (NHLP) and Nif11-related protein (N11P) leader sequences to nitrile hydratase (NHase)

Accession	Description	Motif 1	Motif 2	Leader	Suffix
TIGR01847	Gram-positive leader	N/A	**ELS**EKE**L**AQII**GG**	23	41
TIGR03898	lichenicidin leader	**I**IR**AW**K**DPE**YRAS**L**SSE	**ELSDE**E**L**ESIT**GG**	47	30
TIGR03798	N11P	N/A	**ELSDE**E**L**EAVA**GG**	69	23
TIGR03793	NHLP	**I**KK**AW**S**D**E**EF**KQA**LL**NN	**ELSDE**Q**L**DAVA**GG**	87	27
TIGR03795	NHLP-Burk	**I**AL**AW**H**DPEF**RDE**LL**AD	N/A	108	11
TIGR01323	NHase	VAK**AW**V**DPEF**KAR**LL**KD	G**LS**E**E**Q**L**AALVTR	193	16

Sequence similarities and motif positions in NHase alpha subunit and putative leader sequences. Residues shared by at least 70% of the calculated consensus sequences are shown in boldface. Leader peptide length is taken as the position of the C-terminal end of motif 2, averaged after the shortest 10% and longest 10% removed of each family are removed as outliers and possible gene call errors. The similarity of NHLP to N11P in motif 2 suggests intragenic recombination.

### Non-thiazole/oxazole modified NHLPs

Besides the aforementioned case of *Azospirillum*, additional NHLP family members were found adjacent to a LanM-like lanthionine synthase, instead of a cyclodehydratase-docking fusion protein, in *Nostoc sp*. PCC 7120* and *N. punctiforme *PCC 73102 (* shown in Figure 2, lower panel). LanM is a bifunctional enzyme, responsible for both the dehydration of Ser/Thr residues to dehydroalanine/butyrine and, subsequently, intramolecular Michael-type addition of a Cys thiol to yield (methyl)lanthionines [15,23,51]. Aligning members of this family revealed that sequence conservation is strong over nearly 90 amino acids, and ends with a typical leader sequence cleavage motif, Gly-Gly (Figure 5) [18]. Reminiscent of the TOMM-type NHLPs, the sequence C-terminal of the Gly-Gly motif is short (average length 26) and highly variable. Although not depicted in Figure 5, over 60% of the NHLPs adjacent to LanM-like proteins contain Cys in their core peptide, meaning that these substrates are capable of containing lanthionine crosslinks. Non-TOMM NHLPs lacking Cys in the core peptide will presumably remain at the dehydrated state, unless new tailoring modifications are discovered that further process these groups.

### Post-translationally modified microcins derived from a putative nitrogen-fixing protein

A third protein family, TIGR03798, reprises many of the features of NHLP (Table 1) but are only found in bacteria known to fix nitrogen, with most members also being photosynthetic. TIGR03798 comprises a subset of the Nif11 family (PF07862), which is heavily skewed to the cyanobacteria. Nif11 proteins have no known function [33]. TIGR03798 family members, such as NHLP, occur in fairly large paralogous families. From this point on, we will refer to TIGR03798 as Nif11-derived peptides (N11P). N11P substrates are adjacent to the cyclodehydratase-docking scaffold fusion protein in *C. luteolum *(Figure 2) and nearby in *P. thermopropionicum*. In many cases, N11Ps are adjacent to ABC transport clusters (as defined by TIGR03794, TIGR03796, and TIGR03797) in *C. luteolum*, *Synechococcus sp*. WH 7803, *C. phaeobacteroides*, *Desulfitobacterium hafniense *and *Eggerthella lenta *DSM 2243, among others. Additional N11P members occur adjacent to LanM-like lanthionine-forming enzymes in numerous species of cyanobacteria, including *N. punctiforme *PCC 73102, *Nostoc *sp. PCC 7120, *Prochloroccocus marinus *sp. MIT9313, and *Cyanothece *sp. PCC 7425 (Figure 2) [52]. In the case of *N. punctiforme *PCC 73102, which also possess eight NHLP type substrates (Figure 5), four LanM-like enzymes (Npun_R3205, Npun_R3312, Npun_AF076, and Npun_F5047) are expected to process an additional eight N11P substrates for a total of 16 unique post-translationally modified microcins.

**Figure 6:**
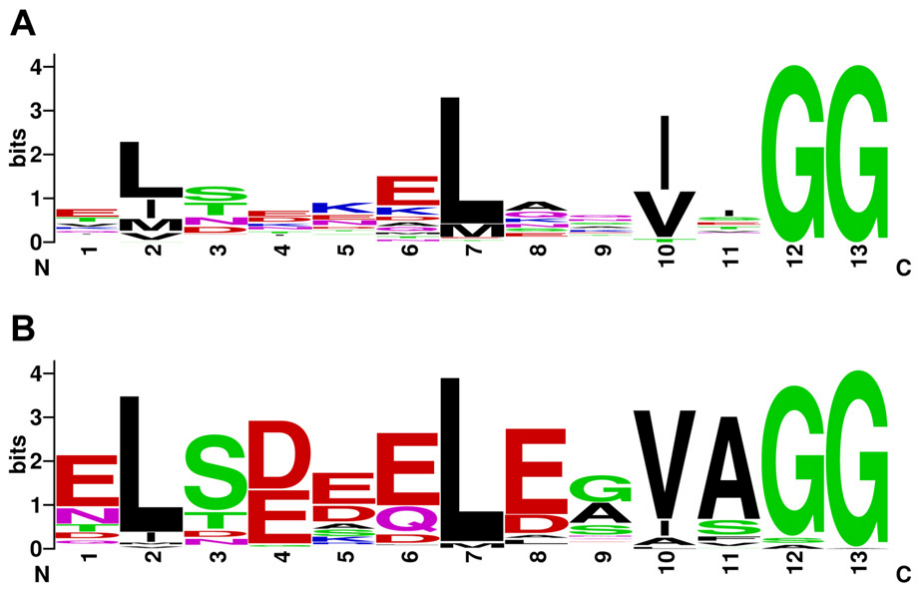
**Sequence logo comparison of classical Gram-positive bacteriocin and nitrile hydratase leader peptides (NHLP)/Nif11-derived peptides (N11Ps)**. (A) Logo depiction of TIGR01847, which is representative of the sequence found near the peptide cleavage site of Gram-positive bacteriocins. (B) Logo for the NHLP and N11P families taken together. In addition to the classic Gly-Gly cleavage motif (positions 12-13), the two logos show an abundance of acidic residues at positions 1, 4 and 6 and a significant preference for Leu at position 2 and 7, Ser/Thr at position 3, and either Val/Leu at position 10. This figure was generated online using a published algorithm [53].

Occurrence in the same genome with a LanM homolog, although not necessarily clustered, is a feature of N11P family proteins from *Synechococcus *sp. RS9916 and *Sinorhizobium medicae *WSM419. Like NHLP and NHLP-Burk, N11P sequences also have a classic leader peptide cleavage motif, usually Gly-Gly, which marks the end of family-wide similarity and the beginning of a low-complexity region rich in Cys, Gly and Ser. As depicted by a logo diagram (Figure 6) [53], the regions leading up to the Gly-Gly motif in NHLPs and N11Ps are quite similar to that of the leader peptides of family TIGR01847 (Table 3), which includes plantaricin A and lactococcin B [41,54,55], two well-known, class II bacteriocins (unmodified microcins).

### Interfamily relationships of NHLP, NHLP-Burk and N11P

None of the three types of transport genes (Trans, Trans-Cleave, Trans-Fuse) identified by PPP have a close homolog in species with NHLP-Burk family members. This implies that the export mechanism, if any, must differ. The occurrence of NHLP-Burk members in pairs, fused in some genomes, suggests a two-chain structure. If exported, these metabolites will likely require a different transport mechanism. The NHLP and NHLP-Burk families do exhibit extensive sequence similarity (motif 1, Table 3), although not in the putative leader peptide cleavage region (motif 2, Table 3). N11P does not show clear evidence of direct similarity to the NHase alpha subunit, as evidenced by extremely poor *E*-values (>1.0) when querying all NHases against any N11P family member. Nevertheless, N11P does exhibit regions of local sequence similarity to NHLP (motif 2, Table 3). To validate the similarity, TIGR03793 (NHLP) and TIGR03798 (N11P) were each searched against species that were known to only contain members of the other family. For instance, a TIGR03793 search against the draft genome of *Synechococcus *sp. RS9916, which contains 31 N11P sequences but no identifiable NHLP sequences, revealed that 19 of the 24 nearest matches are actually members of the N11P family. A similar search performed on *Cyanothece *sp. PCC 7425 returns 13 members of N11P as the top scoring 15 sequences. Such searches also work with members of the NHLP-Burk family. To illustrate, a search with N11P against *Burkholderia *returns a member of NHLP-Burk as the top hit. This cross-specificity, although occurring at the 'noise' level, which is well below the manually set trusted cutoff of each model, reflects two regions of significant similarity between the three precursor families. The more striking region, designated motif 2 (Table 3), is the 13 amino acid stretch leading to the Gly-Gly motif, similar to the leader peptide cleavage region of model TIGR01847. In more classic lantibiotics, such as lacticin 481, similarity of this region to class II bacteriocins has been previously noted [56]. Another region also shows strong sequence similarity between NHLP, NHLP-Burk and N11P. This region, designated motif 1, corresponds to the conserved sequence in the NHase alpha subunit N-terminal to the active site Cys residues (Figure 3). These results, in conjunction with the noted paralogous duplication, are almost certainly the result of intragenic recombination [57].

## Conclusion

The proposed precursor families described in this report dramatically expand the current repertoire of ribosomally produced natural products. This revision includes hundreds of peptides that exhibit (*i*) long leader peptide regions, (*ii*) similarity to proteins and enzymes assigned to other functions and (*iii*) locations distant to the genomic regions used to encode their modification and export genes. Microcins recognized by TIGR01847 have leader peptides predicted to end at an average length of 24 amino acids. However, the corresponding Gly-Gly motifs in the new discovered families presented here end at an average position of 83 and 70 for NHLP and N11P, respectively. NHLPs demonstrate significant sequence similarity to the alpha subunit of NHase, suggesting strongly that they share a common ancestor. NHase is an enzyme with a function unrelated to microcin production and, thus, a broader implication of our findings is that a small protein cannot be automatically excluded from classification as a precursor to a natural product, even if it is homologous to a protein with a known function.

The success of the approach employed here implies that a parallel strategy could prove useful to unravelling other natural product biosynthetic pathways. Possible applications are found in eukaryotic systems, such as in plants, where complex natural product pathways exist, but the requisite genes are not clustered. Clearly, the discovery of new ribosomally produced natural products is far from complete. Even within the families reported here, some members of NHLP and N11P occur in species without identified TOMM or lanthionine-forming enzymes. Furthermore, numerous TOMM clusters remain orphans, with candidate precursors yet to be identified. New tools and concepts, such as those described here, will be of importance in further defining the chemical genetic scope of ribosomally produced natural products.

*Note*: While this manuscript was under review, an independent report was published describing the *in vitro *reconstitution and *in vivo *production of numerous N11P-derived natural products from *P. marinus *sp. MIT9313 [58]. This finding strongly suggests that our informatics-based predictions will hold up to further experimental validation.

## Methods

### General

Multiple sequence alignments were generated using MUSCLE [36] or ClustalW [44], inspected, and refined manually. Refinements included trimming, removal of truncated and other defective sequences, recruitment of additional sequences, and realignment as necessary to create representative seed alignments. Completed seed alignments were used to construct HMMs. The resulting new HMM-based protein family definitions, described in this work, were deposited in the TIGRFAMs database [59,60]. All HMM accessions refer to TIGRFAMs release 9.0 or Pfam release 22 [61].

In order to model regions of local sequence similarity between different protein families, multiple alignments were first generated, trimmed and used to train HMMs for searches to gather additional candidate sequences through an iterated, manual process. HMM construction was performed with the Logical Depth 1.5.4 package software-accelerated emulation of HMMER 2.3. The resulting motif models, of lengths 17 and 13, were searched against the individual families TIGR01323, TIGR03793, TIGR03795, TIGR03798 and the set of 20 proteins that resulted from PSI-BLAST [62]. The PSI-BLAST iterations were carried out to convergence, starting from the predicted 49-residue leader peptide of a hypothetical lanthionine-containing peptide, gi|228993822 from *B. pseudomycoides *SDM 12442), using composition-based statistics and an *E*-value of 0.5. This search strategy provides a working definition for the set of lichenicidin-related bacteriocins homologous in the leader peptide, rather than the core peptide. All non-identical sequences scoring above 0 bits to the respective motif HMMs were aligned to the HMM, resulting in gapless alignments. For each of these, a final HMM was built in order to emit a consensus sequence.

### Description of TIGR (The Institute for Genome Research) models to locate biosynthetic genes

Previous work has identified many cyclodehydratase, dehydrogenase and docking scaffold genes [4,24,27]. In alpha/delta-proteobacteria, actinobacteria, cyanobacteria, and chlorobi type bacteria, the cyclodehydratase and docking scaffolds tend to be found encoded as a single ORF, while other taxa usually produce separate protein products [4]. TIGR03604 describes the docking protein in both fused and unfused cases. TIGR03603 identifies cyclodehydratases that occur as separate genes adjacent to the docking scaffold gene, but a new model, TIGR03882, had to be developed to reliably identify the cyclodehydratase region of the enzymes fused to the docking scaffold. All regions identified by TIGR03882 are fused to a docking scaffold domain, and iteration by PSI-BLAST demonstrates, as expected, weak similarity to a set of known proteins: ThiF of thiamine biosynthesis [63,64], MoeB of molybdopterin biosynthesis [65], ubiquitin E1 conjugating enzymes and the cyclodehydratases identified by TIGR03603. The sequence similarity between post-translationally modified microcins and thiamine/molybdopterin biosynthetic proteins have been previously documented [66]. MccB, an enzyme involved in microcin C7 biosynthesis, also shares considerable similarity to ThiF/MoeB/E1. The Walsh and Schulman groups have recently characterized the MccB protein, confirming the earlier report [67,68]. TIGR03882 recognizes the cyclodehydratase domains of the TriA protein for trichamide biosynthesis in *Trichodesmium erythraeum *[7] and the PatD protein of patellamide biosynthesis in *Prochloron didemni *[8]. The corresponding cyanobactin-type TOMM precursors of these systems are recognized by TIGR03678 [67,68]. Succinct descriptions of all TIGR models of interest to this study are tabulated in Tables 1 and 3.

An examination of the genes in the vicinity of orphan cyclodehydratase-docking scaffold fusion proteins revealed no examples of short peptides qualitatively similar to those previously presented by Lee and Mitchell *et al*. [4]. Previously identified peptides featured leader sequences of approximately 25 amino acids, followed by regions of very low complexity, often of a repetitive nature, and highly enriched in cysteine, serine and threonine. However, our latest survey identified somewhat larger peptides nearby which warranted further investigation as potential TOMM precursors. For each family, founding members were aligned in order to build HMMs and search results were manually inspected in order to set cutoffs for each family. The three families, now represented by TIGRFAMs models TIGR03793, TIGR03795 and TIGR03798 (Table 1) serve as the basis for this report.

### Partial phylogenetic profiling

Selected TIGRFAM models were searched against a collection of 1450 complete or nearly complete bacterial and archaeal genomes. All genomes with at least one protein scoring above the trusted cutoff of the model were assigned the value 1 ('YES') in the phylogenetic profile built to represent that model, while all other genomes were assigned the value 0 ('NO'). By PPP [39], the phylogenetic profile serves as a query to find which genes in a genome may belong to protein families that can best match that profile. PPP produces a score for each protein in a genome by exploring increasing depths in the list of best BLAST matches to that protein. PPP also records the growing set of genomes from which those protein matches originate. At each depth, PPP counts the numbers of genomes agreeing ('YES') and disagreeing ('NO') with the query profile and uses the binomial distribution to score the odds of obtaining at least that many agreements. The overall score for each protein is based on a depth for which the negative log_10 _of the score is maximized, corresponding to an optimum for the working size of a candidate protein family. Each phylogenetic profile was used to query all genomes assigned as YES in the profile. Top-scoring proteins were identified for further analysis. In essence, PPP makes it possible to detect a protein family that matches a query profile, even if that family has never previously been defined.

## List of abbreviations

ABC: ATP-binding cassette; ATP: adenosine triphosphate; HMM: hidden Markov model; *indel*: insertion-deletion; N11P: Nif11-derived peptide; NHase: nitrile hydratase; NHLP: nitrile hydratase leader peptide; NHLP-Burk: *Burkholderia *type TOMM substrate family; Nif11: nitrogen fixation protein of unknown function; ORF: open reading frame; PPP: partial phylogenetic profiling; TIGR: The Institute for Genomic Research; TOMM: thiazole/oxazole-modified microcin.

## Authors' contributions

DHH conceived the study and constructed the TIGRFAM models. DAM participated in the model validation and expanded the scope of the study. MKB developed improved software tools for detecting protein family relationships. DHH and DAM analysed and interpreted the data. DHH and DAM wrote the paper. All authors read and approved the final manuscript.
